# Study on the use of 3D printed guides in the individualized reconstruction of the anterior cruciate ligament

**DOI:** 10.1186/s12891-024-07234-2

**Published:** 2024-02-09

**Authors:** Xin Wang, Dening Wang, Chenchen Zhang, Kefan Zhang, Changling Du, Hui Shi

**Affiliations:** 1 Department of Bone, Nanyang Central Hospital, Henan, China; 2 Department of Bone and Joint, Binzhou Medical University Hospital, Shandong, China

**Keywords:** Anterior cruciate ligament, Anatomical positioning, Guide, Anatomical reconstruction, 3D printing, 3D reconstruction, Anterior cruciate ligament reconstruction, Individualization, Femoral positioning, Gait analysis

## Abstract

**Objective:**

Evaluation of the accuracy and effectiveness of 3D printed guides to assist femoral tunnel preparation in individualised reconstruction of the anterior cruciate ligament.

**Methods:**

Sixty patients who attended the Affiliated Hospital of Binzhou Medical College for autologous hamstring single bundle reconstruction of the anterior cruciate ligament from October 2018 to October 2020 were selected and randomly divided into two groups, including 31 cases in the 3D printing group (14 males and 17 females, mean age 41.94 ± 10.15 years) and 29 cases in the control group (13 males and 16 females, mean age 37.76 ± 10.34 years). Patients in both groups were assessed for intraoperative femoral tunnel accuracy, the number of intraoperative positioning and the time taken to prepare the femoral tunnel, the length of the anteromedial approach incision, the pre-planned bone tunnel length and intraoperative bone tunnel length in the 3D printed group, IKDC score and Lysholm score preoperatively and at 3, 6 and 12 months postoperatively, the Lachman、pivot-shift test preoperatively and at 6 months postoperatively, gait analysis to assess internal and external rotation in flexion of the knee at 12 months postoperatively and postoperative complications in both groups.

**Results:**

There was no statistical difference in functional knee scores and anteromedial approach incision length between the 3D printed and control groups (*p* > 0.05), while there was a statistical difference in the accuracy of tunnel positioning, the time taken to prepare the femoral bone tunnel and the degree of external rotation of the knee in flexion between the two groups (*p* < 0.05). There was no statistical difference between the preoperative planning of the bone tunnel length and the intraoperative bone tunnel length (*p* > 0.05). Complications: One case in the 3D printing group developed intermuscular vein thrombosis in the affected lower limb after surgery, which disappeared after treatment, while three cases in the control group developed intermuscular vein thrombosis in the affected lower limb. No complications such as bone tunnel rupture, deep vein thrombosis in the lower limb and infection occurred in either group.

**Conclusion:**

3D printed guides assisted with individualized ACL reconstruction may improve the accuracy of femoral tunnel positioning, which is safe and effective, while reducing the operative time and the number of intraoperative positioning, without increasing the length of incision, and may obtain higher functional scores and rotational stability of the knee joint, which is in line with the concept of individualized ACL reconstruction.

## Introduction

 Arthroscopic anterior cruciate ligament reconstruction is the accepted treatment for anterior cruciate ligament rupture [1, 2]. Given the high demands and complications of ACL double-bundle reconstruction, anatomically localized single-bundle reconstruction of the ACL is currently more popular [3]. Anatomical positioning for ACL reconstruction restores functional stability to the knee by placing the graft within the original ACL footprint area [4]. Available anatomy [5] demonstrates that anatomical ACL reconstruction can restore anterior-posterior stability and rotational laxity to the cadaveric knee. The data [6] also suggest that current positioning techniques are not sufficient to restore the original ACL centre to the reconstructed graft, resulting in weaker control of knee rotation after ACL reconstruction. Loh [7] et al. claim that grafts placed at 10 o’clock have better control than those placed at 11 o’clock, and therefore recommend a lower position for reconstruction to obtain better rotational stability. Others [8] have suggested that the intercondylar fossa is a three-dimensional structure and that the reference definition of the clock face is not accurate in terms of the location of the tunnel. Jin [9] suggested that the anteromedial bundle also has a role in controlling knee rotation, so positioning the femoral tunnel near the anteromedial bundle stop can restore the original anatomical position and isometricity of the ACL in ACL reconstruction, and this approach has been shown to achieve better clinical results in subsequent studies, but did not quantify the rotation. Another group of scholars [10] advocate the use of Bernard’s four-frame table method for locating the centre of the femoral tunnel. Matthew D [11] studied the kinematic characteristics of the knee joint after ACL reconstruction with different anatomical positioning methods under the same loading conditions by performing ACL reconstruction in cadavers, and found that both the anteromedial technique and the femoral tunnel centrally positioned femoral tunnel resulted in anterior-posterior and rotational stability back to its original functional state. At the same time, the centrally positioned ACL placement further improved the rotational stability of the knee without loss of anterior-posterior stability compared to the centrally placed anteromedial bundle graft. Ideally reconstruction should restore normal knee biomechanics, which requires a patient-specific approach to reconstruction. Individualised reconstruction [12, 13] refers to an anatomical reconstruction to restore the natural ACL function and anatomical position depending on the patient’s anatomy and is currently the most studied form of ACL reconstruction. There is a failure rate of approximately 15% after ACL reconstruction, with technical factors accounting for approximately 24% of these failures [14]. The technical factors 70–80% are deviations in femoral tunnel positioning [14, 15]. Current reconstructive surgical instruments have a single femoral locator that does not cater for the individual differences in lateral femoral condyle anatomy and does not allow for accurate positioning.

The Lysholm [15] and IKDC [16] scores are standardised scores used to evaluate knee symptoms, function and motor activity, and reflect the recovery of knee function in patients. The Lachman test and the axial shift test are important signs of ACL injury and have a high sensitivity [17, 18] and can also be used as indicators of postoperative joint stability.

In order to improve the accuracy of the femoral tunnel, attempts such as Bernard’s four-frame table method under X-ray fluoroscopy [19] and computer-assisted navigation positioning [20–22] have been made, which cannot be promoted in the clinic due to the complexity of the operation or the need for high-end equipment. 3D printing technology can be used to create personalised guides for precise positioning by simulating the anatomical features of the patient’s knee joint. In this study, we collected preoperative knee CT data from patients, carried out preoperative planning, designed and fabricated 3D guides for intraoperative positioning, and investigated the accuracy, safety and effectiveness of 3D printed guides to assist ACL reconstruction. The objective of our study was to evaluate the accuracy and effectiveness of 3D printed guides to assist femoral tunnel preparation in individualised reconstruction of the anterior cruciate ligament.

## Materials and methods

### General information and study population

Sixty patients who attended the Affiliated Hospital of Binzhou Medical College for ACL reconstruction surgery from October 2018 to October 2020 were selected for this study.

The computer randomized into 31 cases in the 3D printing group and 29 cases in the control group, and the preoperative examination and MRI confirmed the diagnosis of ACL rupture with knee instability. The surgery was performed by the same group of surgeons in both groups. The surgical protocol was agreed by the patients and an informed consent form was signed. The study was approved by the Ethics Committee of the Affiliated Hospital of Binzhou Medical College. All patients underwent CT, ECG and laboratory tests upon admission to exclude contraindications.

Inclusion criteria [11–13]: 1. unilateral ACL injury of the knee; 2. grade I or II injury of the meniscus; 3. Outbridge grade I-II injury of the knee cartilage;

Exclusion criteria: 1. combined multi-ligament injury; 2. severe osteoarthritic changes in the knee or severe osteoporosis; 3. combined ACL injury in the contralateral knee; 4. partial rupture of the ACL; 5. patient in growth spurt; 6. severe cartilage injury (Outbridge grade III or IV); 7. meniscus grade III injury; 8. abnormal force lines in the lower limb.

### Pre-operative plan

All patients underwent preoperative thin-section CT (0.625 mm) plain scan of the affected knee to create a three-dimensional model of the knee joint. The femoral tunnel was pre-planned and reserved in the computer software MIMICS (Materialise, Belgium), and the tunnel was positioned using the Bernard four-frame table method [19], i.e. parallel to the direction of the Blumensaat line, at the femur. The distance from the centre of the footprint to the posterior edge of the lateral femoral condyle was approximately 25% of the anterior-posterior edge of the femur, and in the direction perpendicular to the Blumensaat line, the distance between the centre of the femoral footprint and the top of the femoral condyle was 30% of the intercondylar height.

### Design and fabrication of 3D printed guides

**Fig. 1:**
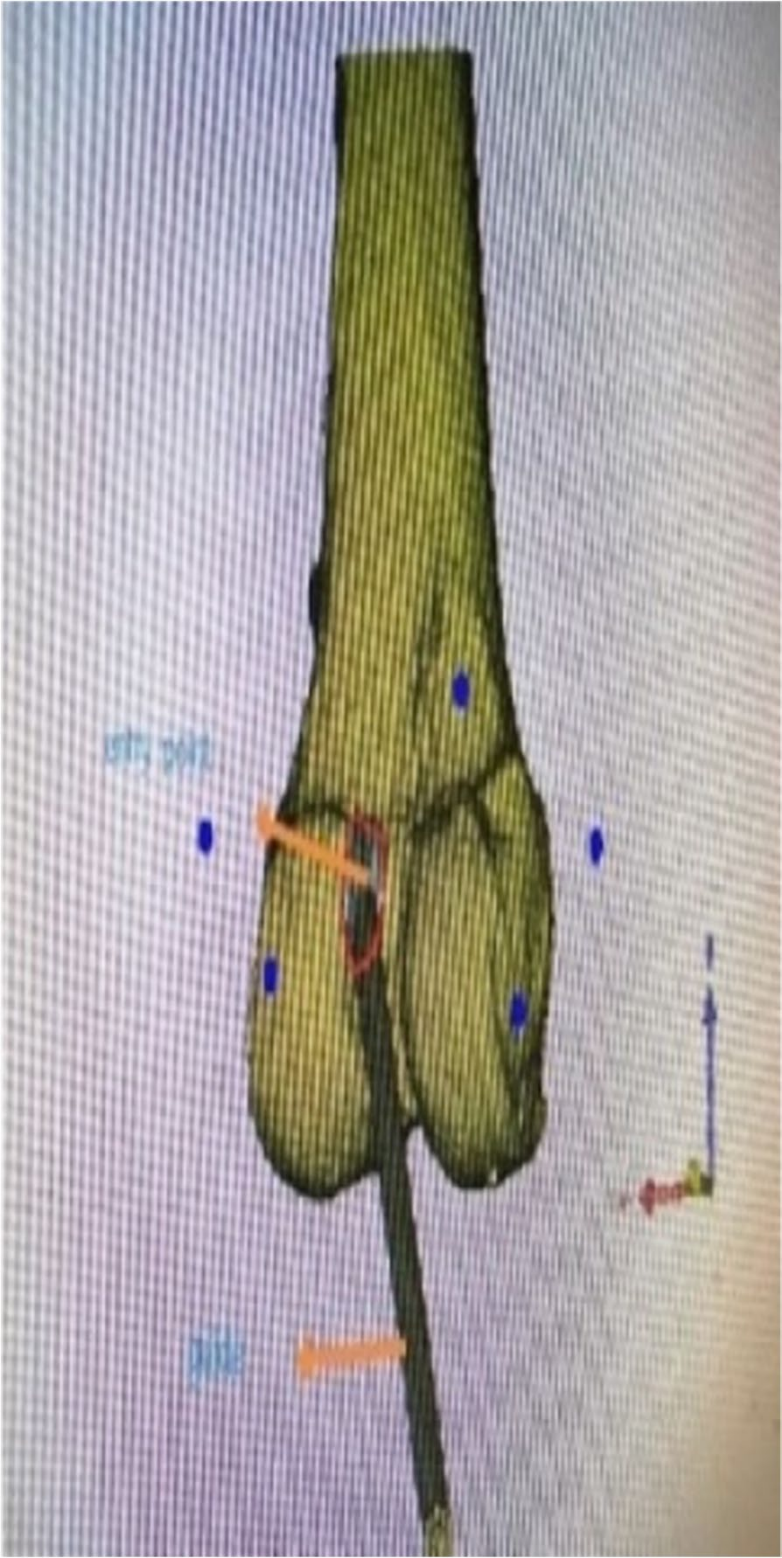
Pre-operative planning: posterior view of a left knee showing the guide entrance at the medial face of the lateral condyle and existing at the lateral femoral cortex

**Fig. 2:**
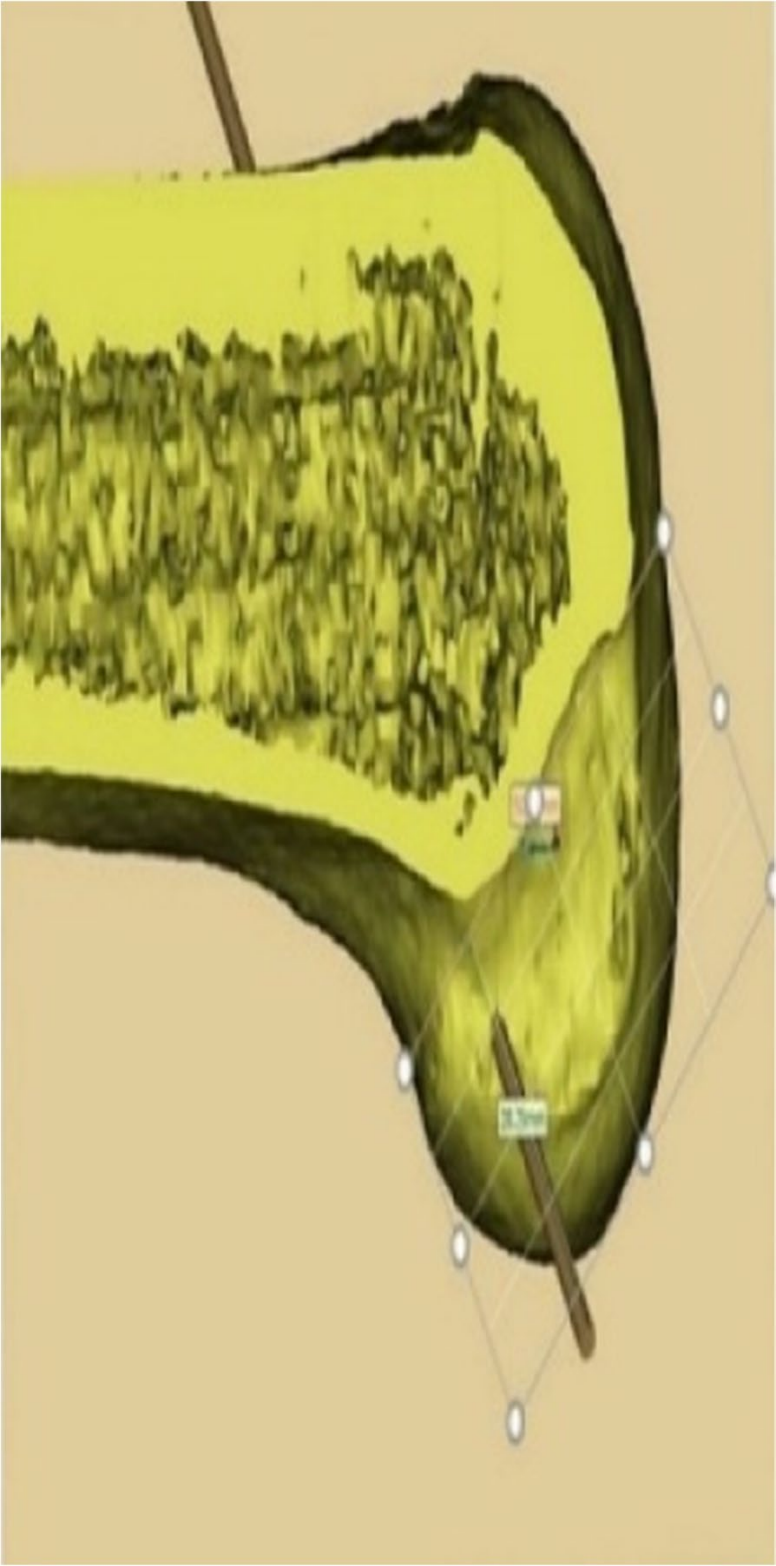
Pre-operative planning: sagittal plane of the left knee showing the guide entrance at the medial face of the lateral condyle and existing at the lateral femoral cortex

**Fig. 3:**
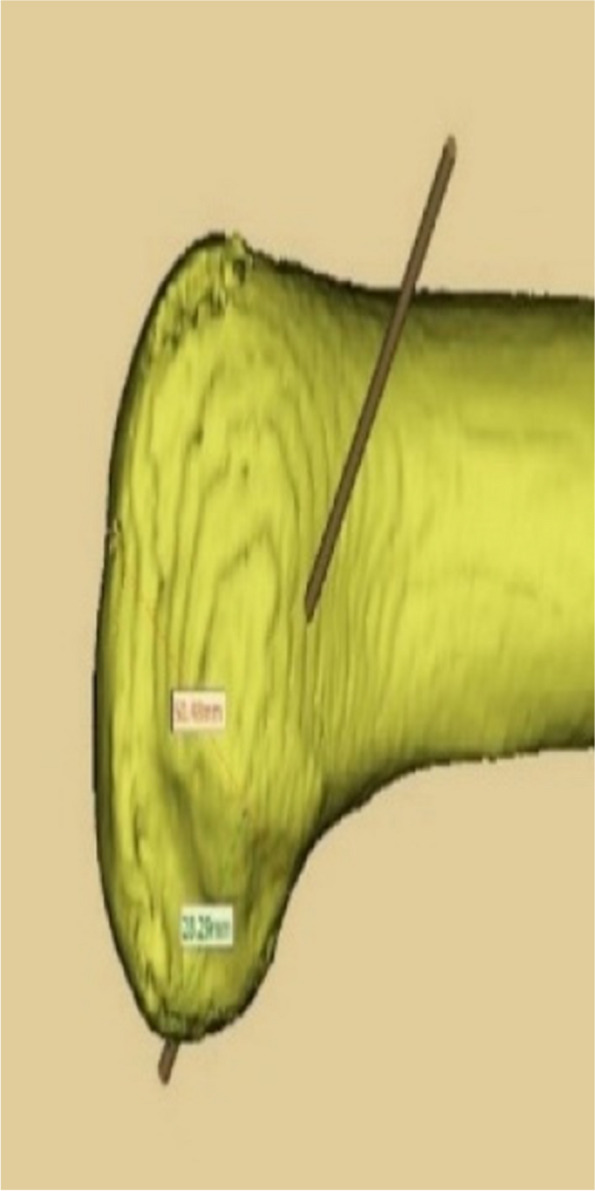
Pre-operative planning: sagittal plane of the left knee showing the guide exit at the lateral face of the lateral condyle and existing at the lateral femoral cortex

**Fig. 4:**
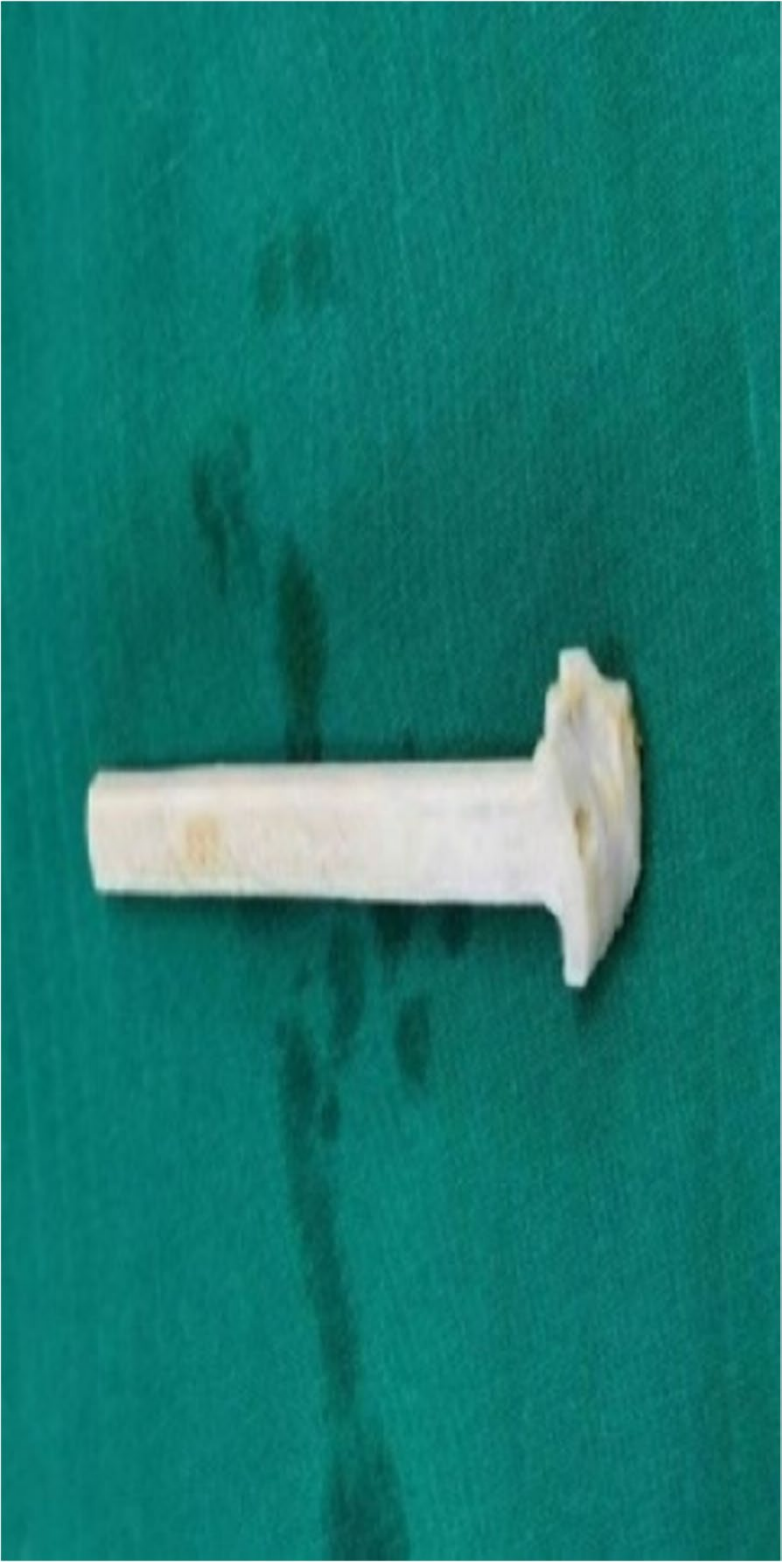
Sterilised spare guide: individualized guide for the left knee made by 3d printing

The CT data of the knee joint of the patients in the 3D printing group were imported into MIMICS, a 3D model was created, and the guides were planned according to the Bernard’s four-grid table method, using the incision between the femoral stem and the posterior aspect of the lateral condyle as the entry point (Figs. 1, 2 and 3). The planned model was printed in STL format on a 3D printer (Allistone, China) using polypropylene resin (PLA), and the guides were sterilised (Fig. 4) and set aside intraoperatively.

### Surgical approach

All patients underwent ACL reconstruction under intravertebral anesthesia and the patient was placed in a supine position with an inflatable tourniquet at the base of the affected thigh. The ACL was explored using a standard anterolateral portal to reconfirm the diagnosis.

#### Graft preparation

**Fig. 5:**
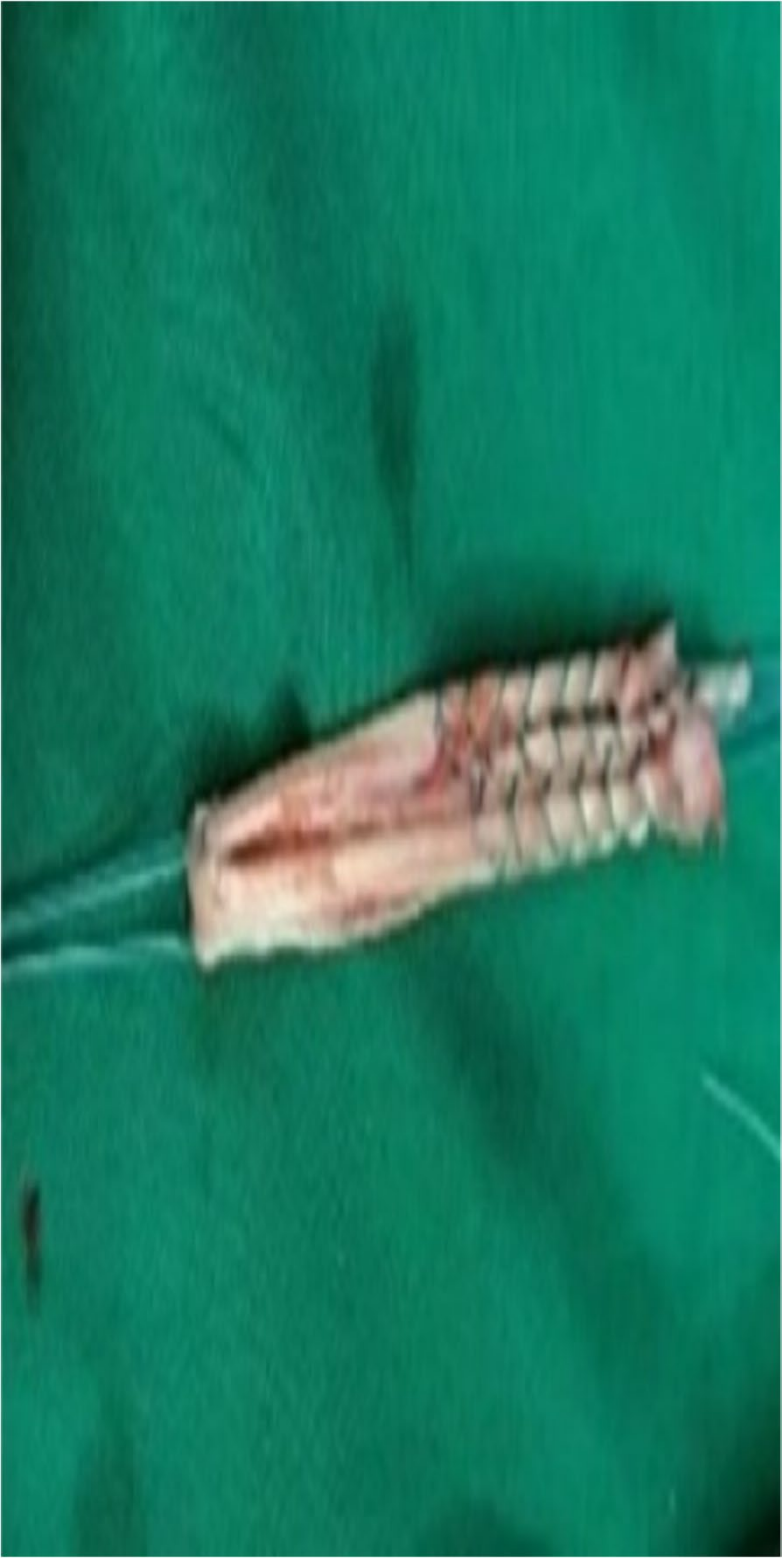
Four strands of autogenous hamstring tendon: examples of prepared tendon

A longitudinal incision of approximately 2–3 cm in length was made 1–2 cm medial to the tibial tuberosity to identify and expose the semitendinosus and gracilis tendons for harvesting tendons and to prepare a four-strand hamstring graft. Each tendon was removed with a closed tendon extractor, they were smoothed out, and removed with a wide bone cutter to preserve the tendinous portion of the muscle. Braided suture with non-absorbable polyethylene sutures were made. The braided tendons was folded symmetrically into four strands with the smallest diameter of the hole through which the tendon can pass (Fig. 5). Pre-drawn and ready for use.

#### Positioning of the tibial tunnel

Both groups are accurately positioned using the ACL tibial guide with the angle adjusted to 55 degrees of flexion. The ACL tibial footprint was located at the midpoint of the line connecting the anterior angle of the lateral meniscus to the medial intercondylar crest, 7 mm anterior to the posterior cruciate ligament, with the outer tunnel opening 2–3 cm below the tibial plateau and 1.5 cm medial to the tibial tuberosity. The tunnel is drilled out with a hollow drill guide pin and the soft tissue surrounding the inner and outer tunnel openings is cleared.

### Creation of the femoral tunnel

#### 3D printing group

**Fig. 6:**
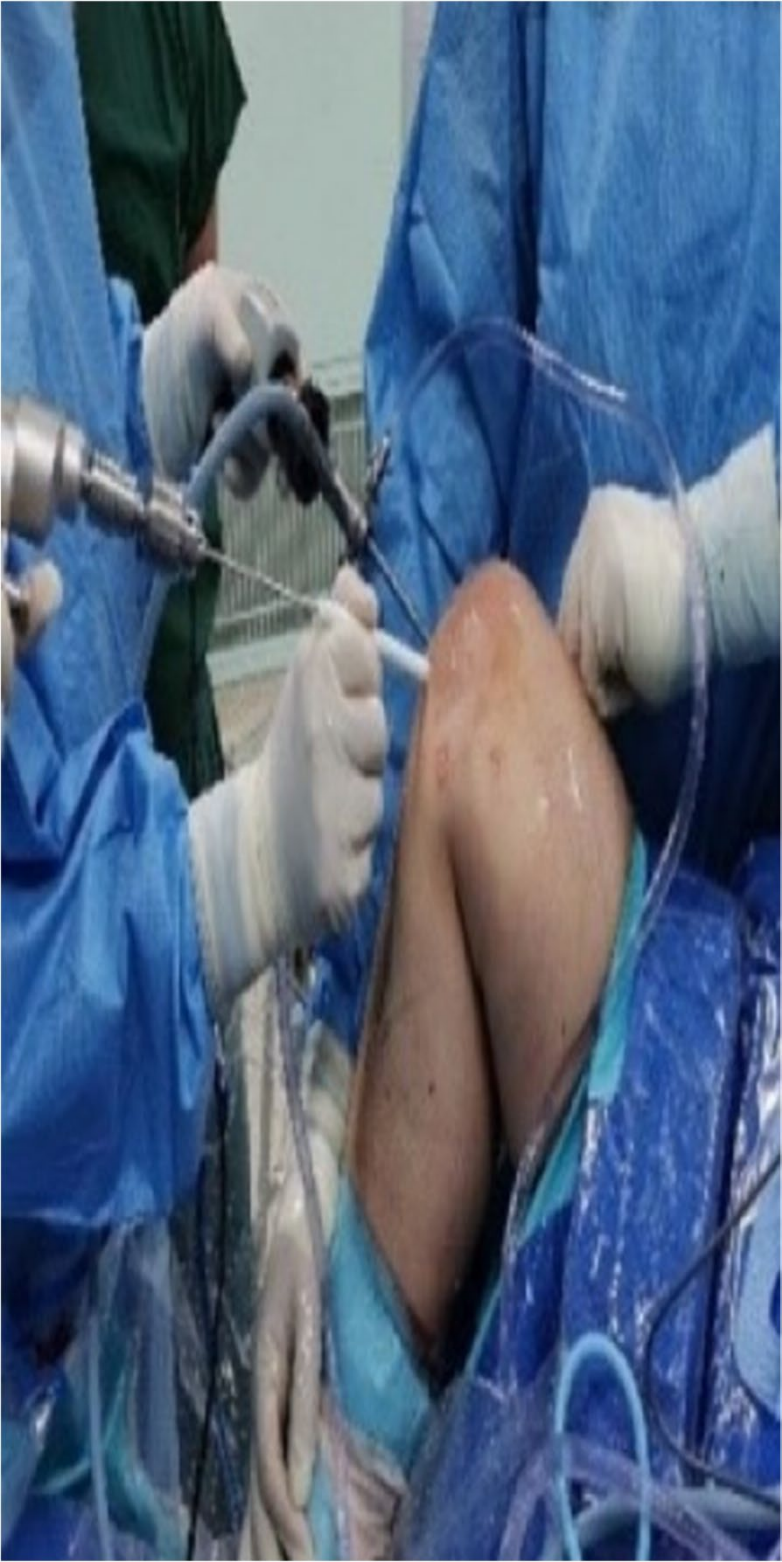
Placement of 3D printed guide arthroscopic femoral tunnel localization using individualized guide

**Fig. 7:**
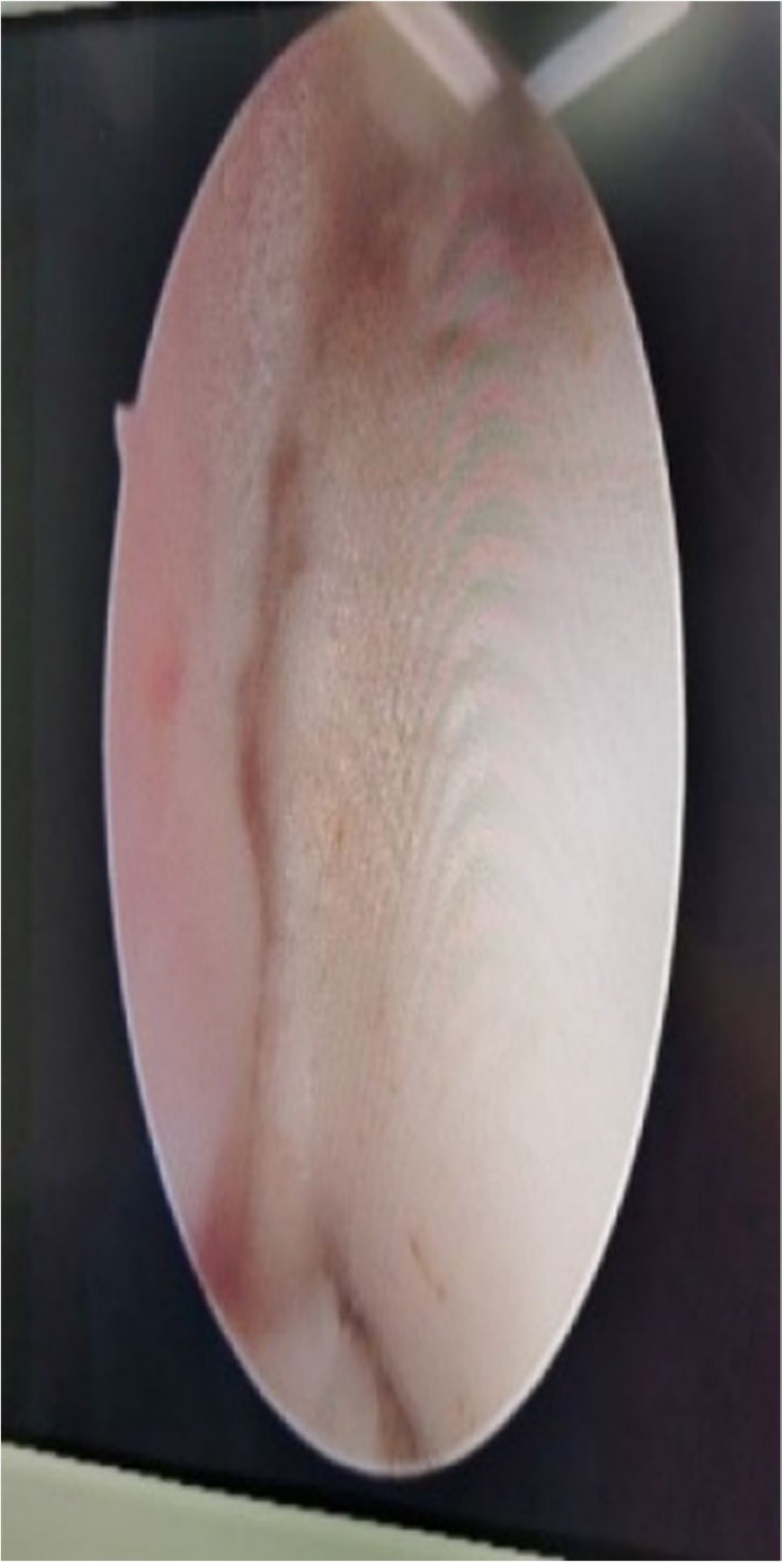
Precise fit of the guide: attachment of individualized guide to the femur in the arthroscopic field of view

**Fig. 8:**
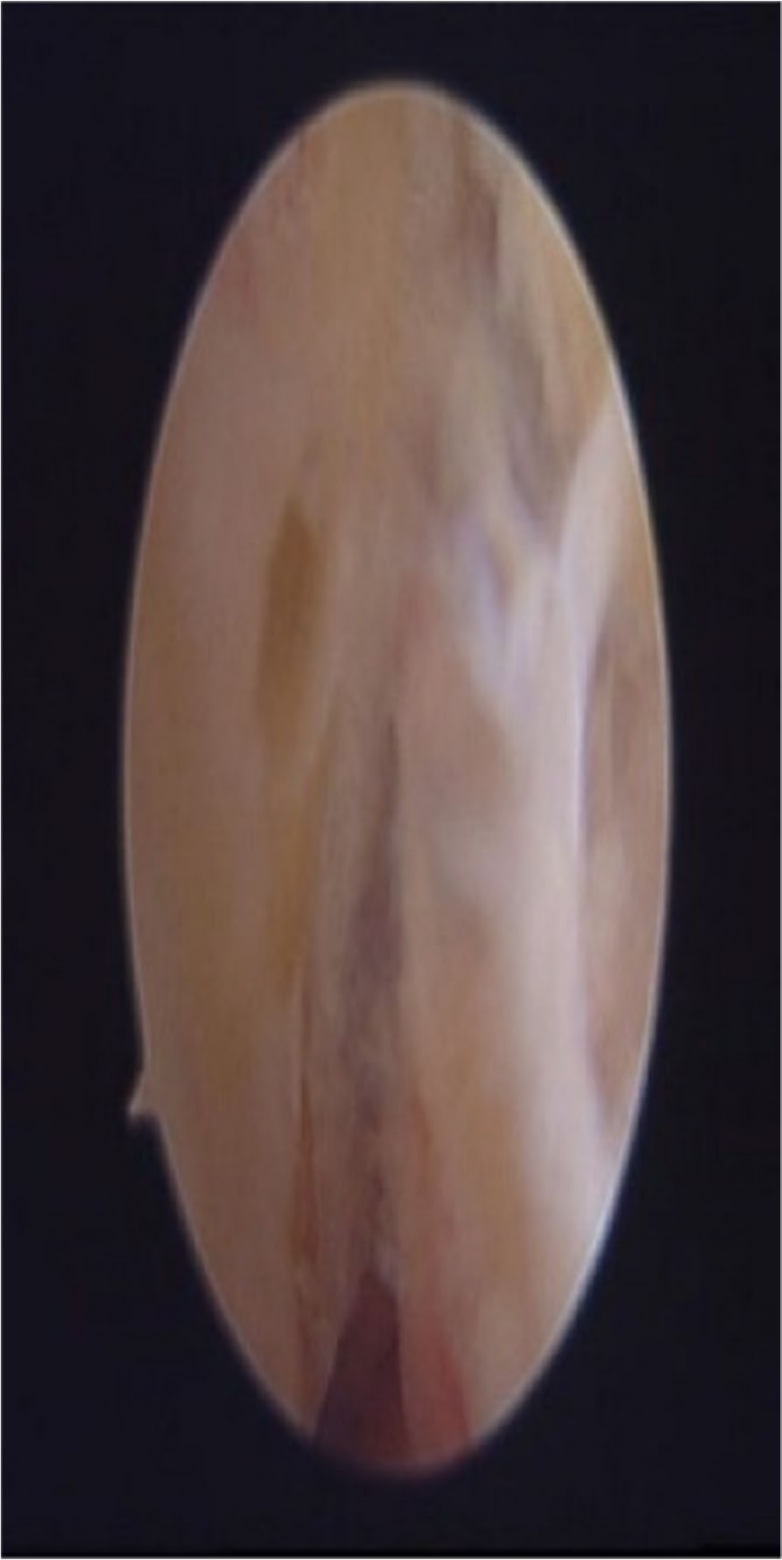
Prepared bone tract: femoral tunnels made by individualized guide under arthroscopic vision

The anterior cruciate ligament insertion site at the femur was adequately cleared and the posterior cartilage of the femoral condyle was completely revealed. The arthroscope was placed at the anterolateral entrance for observation, the patient flexes the knee at 120°, and the guide was placed in the anteromedial approach with the protruding part of the guide placed at the incision to completely fit the bone surface (Fig. 6). The hollow guide was drilled sequentially through the 2.0 mm Kirschner pin and the 4.5 mm hollow drill set to penetrate the contralateral osteocutaneous bone (Fig. 7). The bone tunnel length was measured with a depth measuring tape and the tendon was adjusted according to the length of the bone tunnel and the appropriate plate with collaterals was selected. The bone tunnel created by the 3D printed guide is shown in Fig. 8. (Fig. 8) Selection of titanium plates with fixed length tabs in the right lengths, It means that the length of the tendon in the lateral femoral tunnel is guaranteed to be greater than 20 mm after the use of a fixed-length titanium plate.

#### Conventional group

**Fig. 9:**
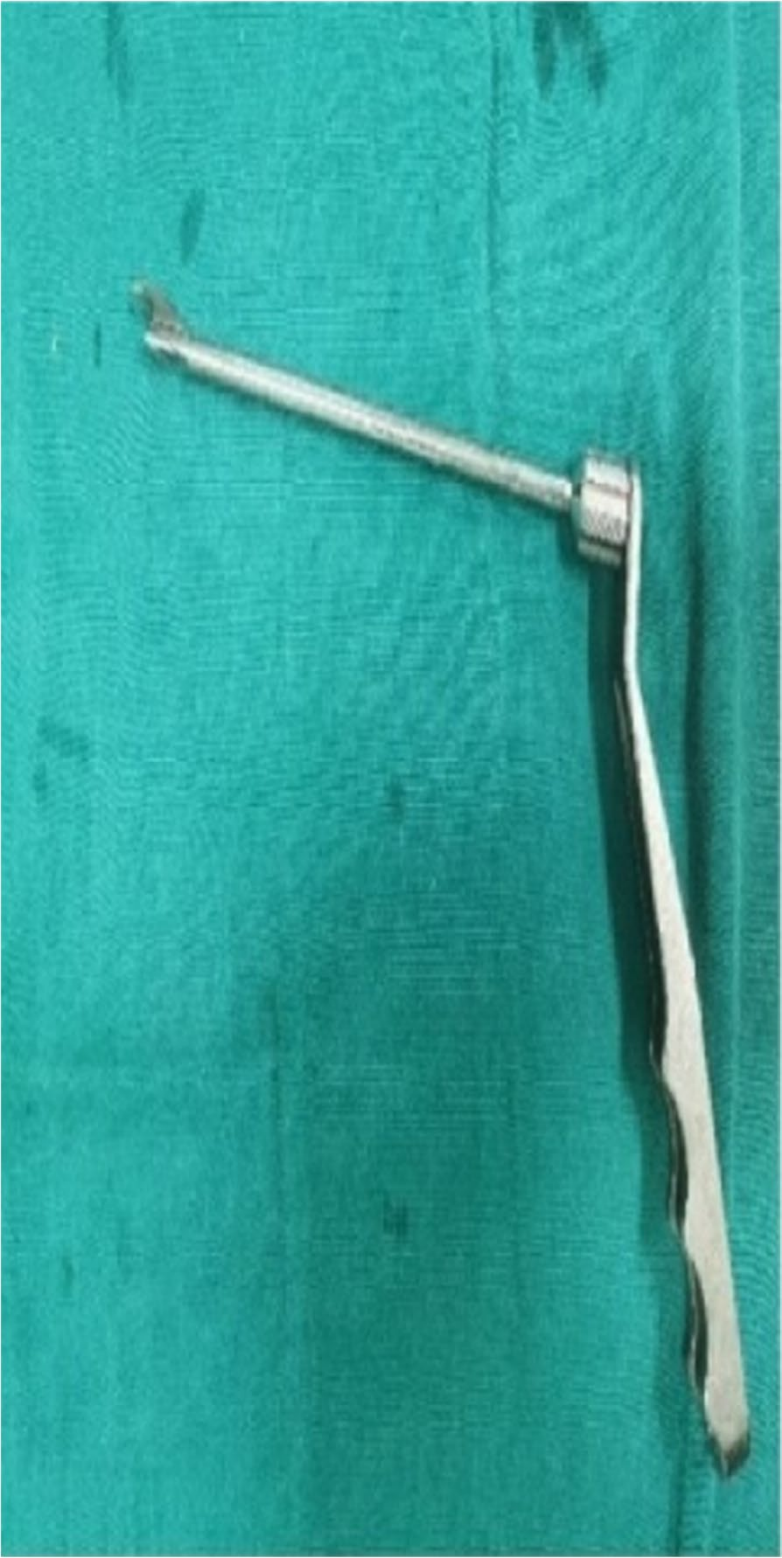
Smith & Nephew fixed guide

**Fig. 10:**
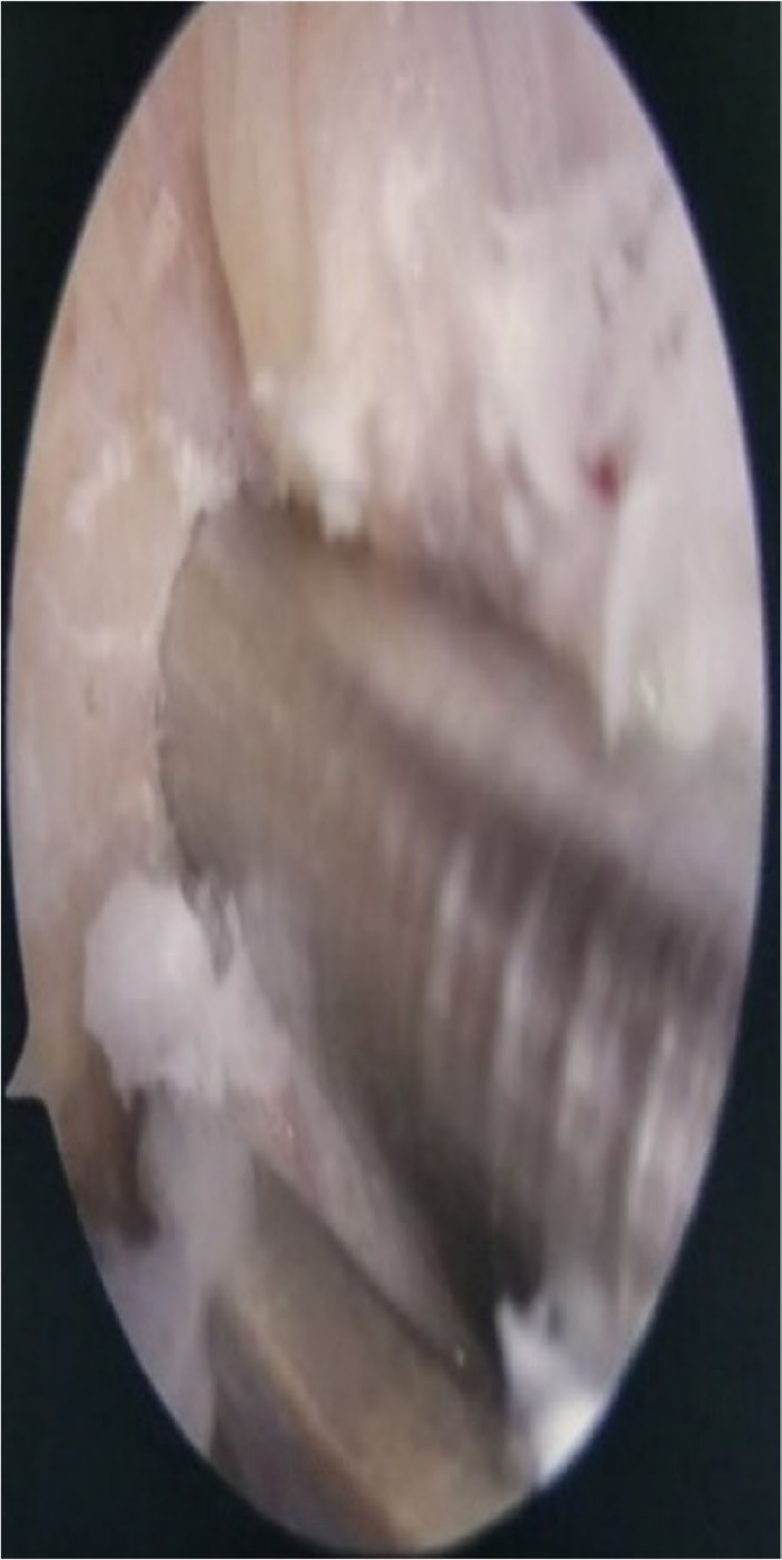
Positioning of fixed guide and tunnel preparation: femoral tunnels made by Smith & Nephew guide in the arthroscopic field of view

The guide was a fixed femoral guide from Smith & Nephew. The guide was selected according to the diameter of the tendon, with the radius of the tendon + 2 mm (thickness of the posterior wall of the tunnel) being the guide type (Fig. 9). After positioning the 2.0 mm Kirschner pin, a 4.5 mm hollow drill was set in to penetrate the contralateral osteocutaneous bone (Fig. 10). A depth gauge measures the length of the bone tunnel and the appropriate plate with tabs was selected according to the length of the bone tunnel.

### Graft fixation

In both groups, the tendon was introduced through the tibial bone tunnel using a guide wire, ensuring 30° of knee flexion, and the femoral side was fixed with a plate system with tabs (the RIGIDLOOP®, DepuyMitek, Johnson & Johnson) and the tibial bone tunnel was fixed with a squeeze screw fixation system (the INTRAFIX® ADVANCE Tibial Fastener System, DepuyMitek, Johnson & Johnson). Arthroscopic re-examination of reconstructed ACL position morphology and tension in extension and no impingement in the intercondylar fossa was verified after graft fixation. The anterior drawer test, Lachman test and pivot shift test were performed to check joint stability again. The incision was closed sequentially after rinsing and haemostasis. Postoperatively, the knee was fixed in a restrictive brace.

### Intraoperative statistics

The number of intraoperative positions, the time taken for femoral tunnel preparation, the length of the femoral tunnel and the length of the anterolateral approach incision were recorded for both groups.

### Post-operative rehabilitation

The same postoperative rehabilitation programme was used in both groups, with quadriceps isometric contraction and ankle pump exercises starting on the day after the surgery. On the second day, the patient was placed on crutches in a brace-extended position and continued these exercises. The quadriceps isometric contraction and ankle pump exercises were continued for one week after surgery, while pushing on the patella to prevent adhesions. The brace, straight leg raising in bed and quadriceps exercises were used for one or two weeks post-operatively. At two or four weeks postoperatively, the patient will walk on crutches without full weight bearing, slowly increasing the range of motion by approximately thirty degrees per week to ninety degrees at four weeks. Six weeks postoperatively, full weight bearing will be attempted and the quadriceps muscle strength will be intensified. Eight weeks postoperatively, normal exercise will be resumed and the joint mobility should be at least one hundred and twenty degrees. From nine weeks to six months after surgery, remove the brace and continue to strengthen the muscles, practise fast running and balance board and proprioceptive training. From sevent months to one year after surgery, you can gradually resume special training or full activity.

### Post-operative follow-up

A CT scan of the knee was performed three days after surgery and the accuracy of the femoral tunnel was compared between the two groups of patients. Patients were followed up at one month, two months, three months, six months and twelve months post-operatively in the outpatient clinic and instructed to perform functional exercises. Knee function scores (IKDC score and Lysholm score) were performed at the follow-up visits in three months, six months and twelve months after surgery, and Lachman test and pivot-shift test were performed on the affected knee six months after surgery. Gait analysis was performed on the patients in twelve months after surgery.

### Statistical analysis

**Fig. 11:**
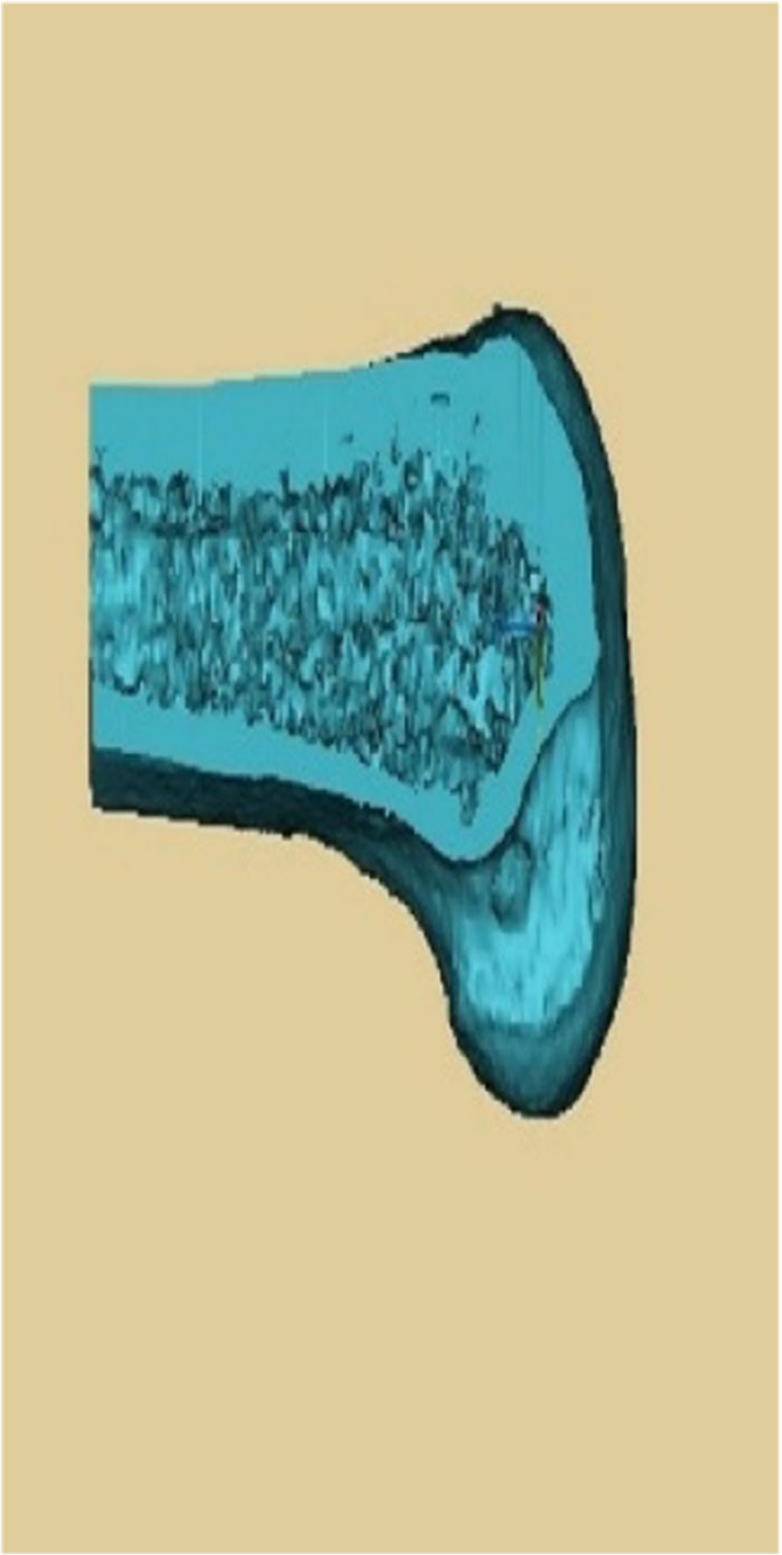
Shows the femoral tunnel on postoperative CT

**Fig. 12:**
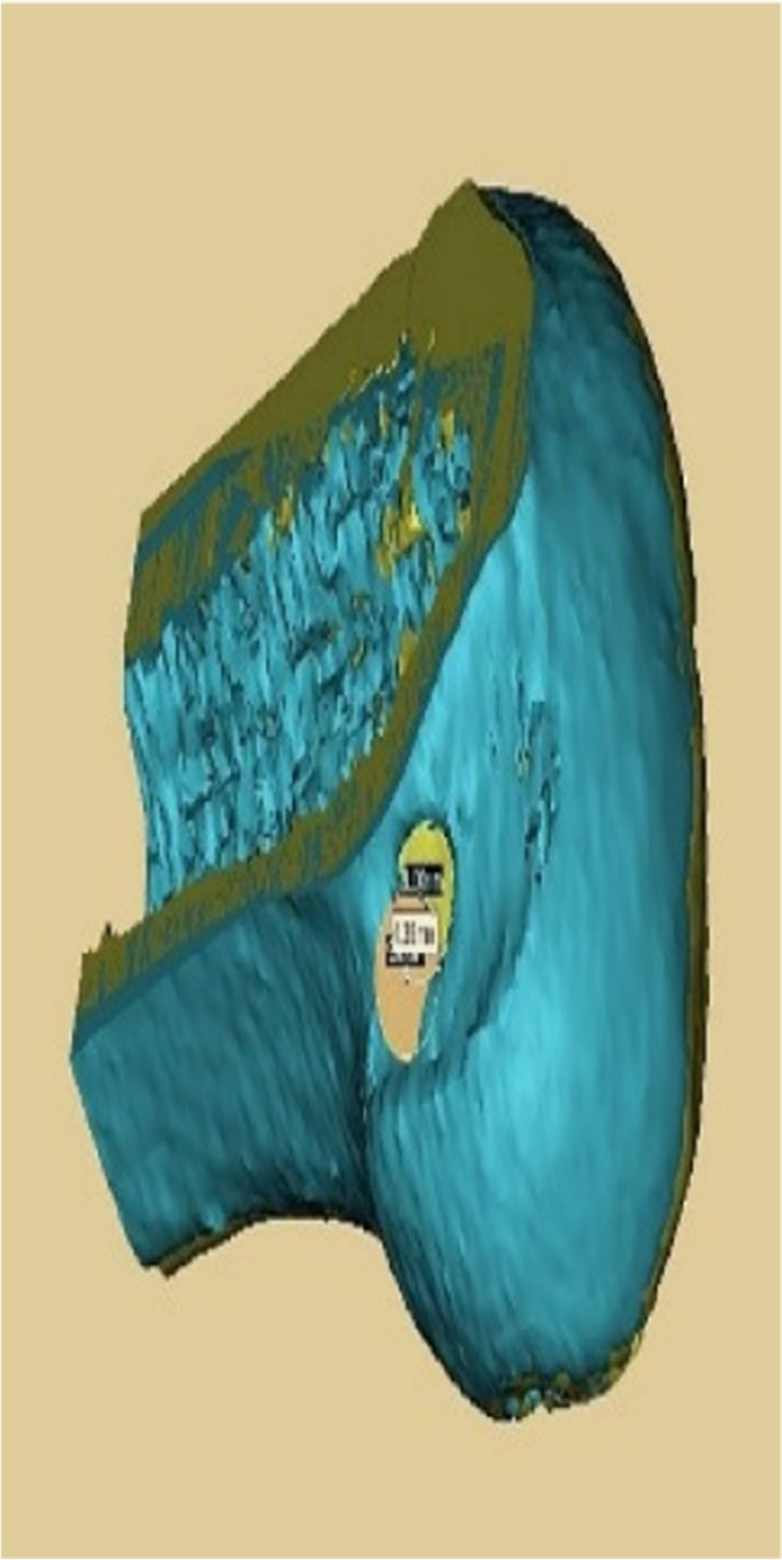
Shows the accuracy measurements (preoperative 3D CT of the knee in yellow, postoperative 3D CT of the knee in blue)

Data were analysed by SPSS 26.0 software. Data on general patient data, pre- and post-operative IKDC and Lysholm scores, number of intraoperative localisations and time taken to prepare the femoral tunnel in both groups, length of the anterolateral approach incision, number of degrees of internal and external rotation in knee flexion, accuracy and length of the bone tunnel were expressed as mean ± standard deviation (x ± s), using independent samples t-test. *P* < 0.05 means that there is a statistical difference. The accuracy of tunnel positioning was based on the junction area of the two tunnels as a percentage of the pre-planned tunnel area when the preoperative and postoperative 3D reconstructions of the knee were fully reunited (Figs. 11 and 12).

## Results

There was no statistical difference in gender, age and in the preoperative Lysholm score and IKDC between the two groups included in the statistics (*P* > 0.05) (see Table 1). One year after surgery, the knee function scores of both groups were significantly higher than those before surgery, and the difference was statistically significant (*P* < 0.05). At the same time point, there was no significant difference in the Lysholm and IKDC scores of the knee joint in the 3D printing group compared to the Control group (*P* > 0.05) (see Tables 2 and 3). There was a statistically significant comparison of the pre- and post-operative Lachman test and pivot-shift test between the two groups (*P* < 0.05), and the post-operative negative rate was significantly higher in both groups (see Tables 4 and 5). There was a significant difference in the time to prepare the bone tunnel and the mean number of times the bone was positioned between the two groups (*P* < 0.05), with the mean time taken to position and prepare the tunnel and the number of times it was positioned being less in the 3D printing group than in the control group. No significant difference was seen in the length of the anterolateral approach incision when comparing between groups (*P* > 0.05) (see Table 6). In the positioning accuracy comparison, the accuracy was 49.57% ± 6.31% in the Control group and 58.18% ± 5.55% in the 3D printing group. (See Table 7) and the difference was tested to be statistically significant ( *P* < 0.05). The results of the postoperative gait analysis showed no restoration of rotational stability of the knee in the short term (12 months) with ACL reconstruction and increased external rotation of the affected knee compared to the healthy side in both the 3D printed and control groups (*P* < 0.05). When comparing between groups in the foot flattening phase and toe off phase, the 3D printed group had less external rotation of the affected limb than the control group (*p* < 0.05) (see Table 8). In terms of bone tunnel length, there was no statistical difference between the 3D group and control groups in terms of intraoperative measured bone tunnel length compared to the pre-operative pre-planned tunnel length (*P* > 0.05) (see Table 9). The femoral tunnel in the 3D printed group was significantly more accurate than the conventional group, with positioning closer to the pre-planned tunnel position and higher repeatability and stability than the control group. In terms of complications, at 1 year follow-up, there were no knee infections or femoral tunnel fractures in either group after surgery. At the same time, the size of the guide was comparable to that of the conventional femoral tunnel locator, and there was no statistical difference in the length of the surgical incision between the two groups.

**Table 1:** Descriptive data of the patients

	Males	Females	Average age	t Value	*P *Value
Control group	13	16	37.76 ± 10.34岁	1.58	0.12
3D printing group	14	17	41.94 ± 10.15岁		
χ^2^ Value	0.001				
*P* Value	0.98				

**Table 2:** Comparison of Lysholm scores before and after surgery in both groups

Group	Pre-operative	Post-operative 3 months	Post-operative 6 months	Post-operative 12 months	Post-operative 12 months vs. pre-operative T Value	Post-operative 12 months vs. pre-operative *P* Value
Control group	48.45 ± 4.66	71.17 ± 5.90	81.07 ± 3.47	91.89 ± 4.88	-36.167	***P*** **< 0.05**
3D printing group	50.13 ± 3.96	72.23 ± 3.51	82.90 ± 3.85	92.45 ± 4.64	-38.650	***P*** **< 0.05**
T Value	-1.509	-0.847	-1.933	-0.471		
*P* Value	> 0.05	> 0.05	> 0.05	> 0.05		

**Table 3:** Comparison of IKDC scores before and after surgery in both groups

Group	Pre-operative	Post-operative 3 months	Post-operative 6 months	Post-operative 12 months	Post-operative 12 months vs. pre-operative T Value	Post-operative 12 months vs. pre-operative *P* Value
Control group	51.07 ± 7.02	72.34 ± 6.41	84.69 ± 3.76	90.93 ± 6.09	-23.092	***P* < 0.05 **
3D printing group	52.77 ± 7.39	73.32 ± 5.78	86.03 ± 4.85	92.5 ± 4.86	-25.101	***P* < 0.05 **
T Value	-0.916	-0.621	-1.192	-1.163		
*P* Value	> 0.05	> 0.05	> 0.05	> 0.05		

**Table 4:** Comparison of control group Lachman and pivot-shift test preoperatively and 12 months postoperatively

Phases	Lachman test		Pivot-shift test	
	Positive	Negative	Positive	Negative
Pre-operative	23 (75.0%)	6 (25.0%)	25 (80.0%)	4 (20.0%)
Post-operative 12 months	4 (5.0%)	25 (95.0%)	2 (10.0%)	27 (90.0%)
χ^2^ Value	25.02		36.66	
*P* Value	***P* < 0.01 **		***P* < 0.01 **	

**Table 5:** Comparison of preoperative and 12 months postoperative Lachman and axis shift tests in the 3D printing group

Phases	Lachman test		Pivot-shift test	
	Positive	Negative	Positive	Negative
Pre-operative	27 (80.0%)	4 (20.0%)	25 (65.0%)	6 (35.0%)
Post-operative 12 months	0 (0%)	31 (100%)	3 (10.0%)	28 (90.0%)
χ^2^ Value	47.83		31.52	
*P* Value	***P* < 0.01 **		***P* < 0.01 **	

**Table 6:** Comparison of the number of positions, preparation time of the tunnel and length of the anterolateral approach incision between the 3D printing group and the control group

Group	Tunnel positioning time (seconds)	Length of anterolateral approach incision (cm)	Average number of positions (times)
Control group	262.31 ± 23.08	1.84 ± 0.95	1.29 ± 0.58
3D Printing Group	210.85 ± 17.73	1.86 ± 0.92	1.69 ± 0.81
T Value	7.91	-7.45	-2.20
*P* Value	***P* < 0.05 **	*P* > 0.05	***P* < 0.05 **

**Table 7:** Comparison of accuracy between the 3D printing group and the conventional group

Group	Accuracy
Control group	49.57 ± 6.31
3D printing group	58.18 ± 5.55
T Value	-4.58
*P* Value	**< 0.05**

**Table 8:** Gait analysis (external rotation) at 12 months after ACL reconstruction

Time phase	Group	Knee rotation degrees		T Value	*P* Value
Foot flattening phase	Control group	23.59 ± 3.75	19.48 ± 5.39	3.32	**0.002**
	3D Printing Group	21.06 ± 5.56	17.61 ± 4.76	2.63	**0.011**
	T Value	2.05	1.41		
	*P* Value	**0.045**	0.163		
Toe off phase	Control group	16.38 ± 6.49	12.19 ± 5.47	2.67	**0.010**
	3D Printing Group	12.58 ± 7.16	10.16 ± 5.26	1.52	0.135
	T Value	2.15	1.45		
	*P* Value	**0.036**	0.153		

**Table 9:** Comparison of preoperative planned bone tunnel length and intraoperative bone tunnel length in the 3D printing group

Group	Length (mm)
Control group	33.19 ± 3.83
3D printing group	34.45 ± 3.55
T Value	-1.34
*P* Value	0.186

## Discussion

With the development of the ACL concept, some scholars [23, 24] now prefer individualised reconstruction, arguing that ACL reconstruction should demonstrate individual anatomical variability in order to restore normal knee motion. In contrast, 3D printed guides can be made to fit the bone surface in conjunction with the patient’s own knee anatomy, reducing mirror image and anatomical differences to a certain extent. Dejian Liu [25] used 3D printing to create a personalised navigation template to assist in ACL reconstruction and found no difference between the postoperative bone tunnel position shown in the 3D group and the pre-planned bone tunnel position (*p* > 0.05). The 3D printed guides also showed good positioning accuracy compared to the conventional ACL reconstruction method. This study required simultaneous MRI scans of the patient’s healthy knee, which increased the patient’s hospital costs, and did not quantify the rotation of the ACL reconstruction. In our study the outcomes were similar. During surgery, some scholars have performed repeated positioning and intraoperative fluoroscopy in pursuit of accuracy and improved postoperative knee function, resulting in reduced strength of the lateral femoral bone, prolonged operative time, and increased risk of femoral tunnel wall fracture and infection [26]. In contrast, this study found that ACL reconstruction by 3D printed guides reduced positioning time and frequency, with better results than the control group, and no complications such as bone tunnel fracture or infection occurred.

In this study, the functional scores of the knee at 3 months, 6 months and 1 year after surgery were significantly higher than the preoperative scores in both groups, and the negative rates of the Lachman test and the pivot-shift test were significantly higher compared to the preoperative scores, indicating that both the 3D-printed and conventional ACL reconstruction groups could achieve satisfactory results in terms of knee stability recovery. This is similar to Dejian Liu’s [25] results. However, the rotational stability of the knee joint illustrated by the Lachman test and the axial shift test are not optimal results and should be further shown by quantification. It has been claimed that the rotational stability of the knee joint is measured by devices such as computerized navigation, which increases the difficulty of related research due to the expensive and non-portable nature of computerized navigation devices. This paper is the first to combine 3D printed individualized guides with gait analysis to investigate knee stability through changes during gait, which is simple and practical and helps to advance clinical work.

The gait analysis was somewhat reflective of the patient’s pathological movement pattern. In extension, the knee is unable to rotate due to the ‘snap-lock’ mechanism and in flexion the knee rotation angle increases significantly. There were two peaks of knee flexion during the entire gait cycle. Statistical analysis of the peak rotation angles showed that during the same gait period, the postoperative external rotation angle was significantly smaller in the 3D printed group compared to the control group. In the control group, the affected knee showed more external rotation than the healthy knee in both the foot flattening and toe off phases. In the toe-off phase, the affected knee showed a comparable amount of external rotation to the healthy side. The gait results suggest that ACL reconstruction may not restore proper rotational stability to the patient and that precise positioning and preparation of the lateral femoral tunnel may help to improve the rotational stability of the patient’s knee. Komzak et al [27], reported a randomized trial that similarly found that anatomical single-bundle ACL anatomical reconstruction was not sufficient to restore rotational stability. Another study [28] evaluated the kinematics of the ACLR knee and the contralateral normal knee during downhill slopes in six patients and found that the affected knee exhibited more external rotation compared to the contralateral knee, which is consistent with our findings, but with a different aim of the study. He [29] performed ACL reconstruction using a personalized femoral positioner-assisted procedure and found that gait analysis of the affected knee 12 months after surgery demonstrated rotational stability that was not significantly different from that of the control group. The demonstrated results were the same as ours. In addition, relevant studies [30] have also demonstrated that computerized navigation can improve the accuracy of tunnel anatomical orientation and position in ACLR surgery, further affecting the rotational stability of the knee. However, fewer studies have been conducted to quantify the rotational stability of the knee joint, either with 3D-printed individualized guides or computer navigation, and the results still need to be confirmed by further investigation.

The difficulty in this study was the determination of the guide plate entry point, the setting of the tunnel position and the orientation. One study [31] claimed that reconstructive ligamentous tendon bone healing is closely related to the length of the tendon within the bone tunnel, which is generally considered to require at least 15–20 mm.In this study, we attempted to preserve a bone tunnel of at least 30–40 mm in the preoperative planning to ensure a long enough bone tunnel for tendon bone healing.

There are shortcomings in the 3D printed guide technique to improve accuracy. The system related to 3D printing technology today is not robust enough, its operation is relatively cumbersome, the learning curve is long and there is waste of materials. At the same time, there are limitations in this study: firstly, we had a relatively short follow-up period and we did not observe the functional status of the knee joint and gait analysis of the patients after 1 year; secondly, we included fewer subjects in this study, which may have biased the results; thirdly, in the gait analysis, we only analysed the effect of ACL injury on the knee joint, ignoring the effect of the hip and ankle joints on the knee joint in flexion.

## Summary

3D printed guides assisted ACL reconstruction may significantly improve the accuracy of femoral tunnel positioning, with low complications, safe and effective, while reducing the operative time and number of intraoperative positions, without increasing the length of the incision, resulting in higher functional knee scores and improved rotational stability of the knee, in line with the concept of individualised ACL reconstruction.

## Data Availability

The datasets used and/or analysed during the current study are available from the corresponding author on reasonable request. If someone wants to request the data from this study, please contact the first Author (Xin Wang, wang1223306@163.com).
